# Supplementary material for: Quantitative Risk Assessment for the Introduction of Bovine Leukemia Virus-Infected Cattle Using a Cattle Movement Network Analysis
Source: Pathogens. 2020 Oct 28;9(11):903. doi: 10.3390/pathogens9110903 (PMC7693104; doi:10.3390/pathogens9110903)
Supplement: Supplementary file 1 [file pathogens-09-00903-s001.zip › Supplementary figures.pdf]

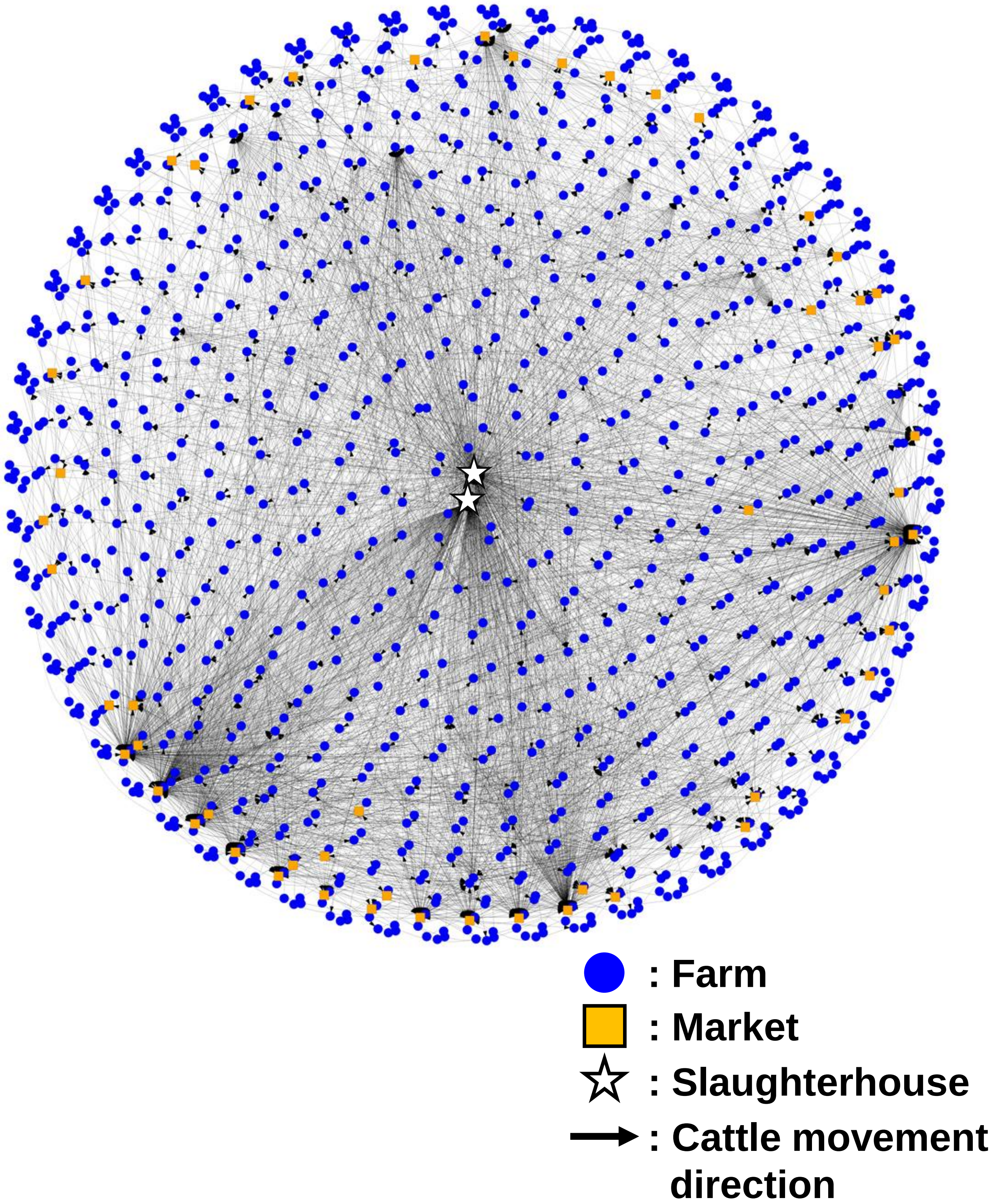

Figure S1. Non-weighted cattle movement network.

In total, 2,963 movements between holdings (farm-farm, farm-market, market-farm, farm-slaughterhouse, market-slaughterhouse) were identified from 1,097 farms, 55 markets, and 2 slaughterhouses. The movement direction of the cattle is indicated by direct edges. Farms, markets, and slaughterhouses are indicated by nodes depicted as blue circles, orange squares, and white stars, respectively.

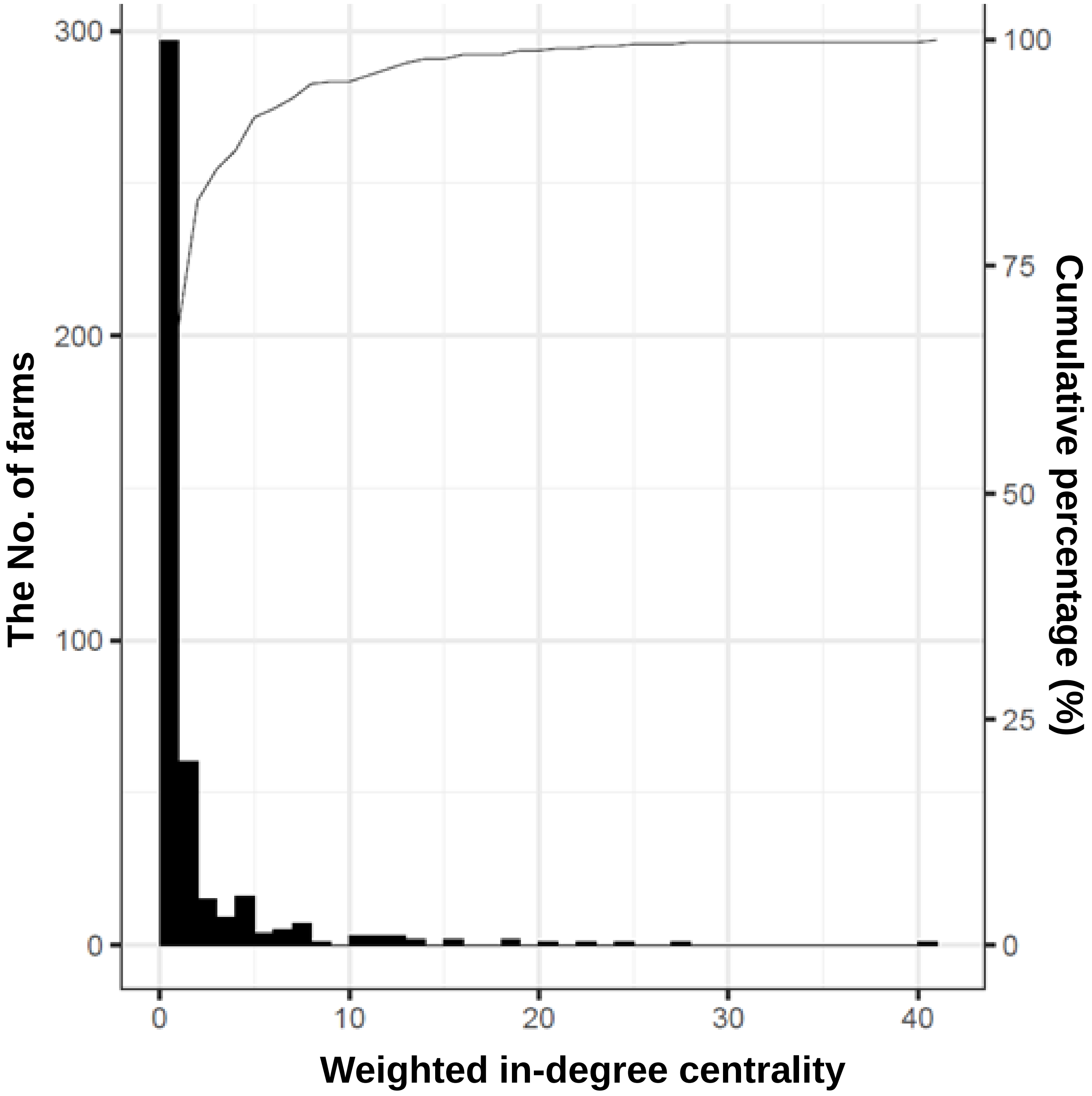

Figure S2. Distribution of the number of farms versus the value of weighted in-degree centrality.

The histogram indicates the distribution of the number of farms versus the value of weighted in-degree centrality. The line indicates the cumulative percentage of the number of farms in relation to the value of weighted in-degree centrality.

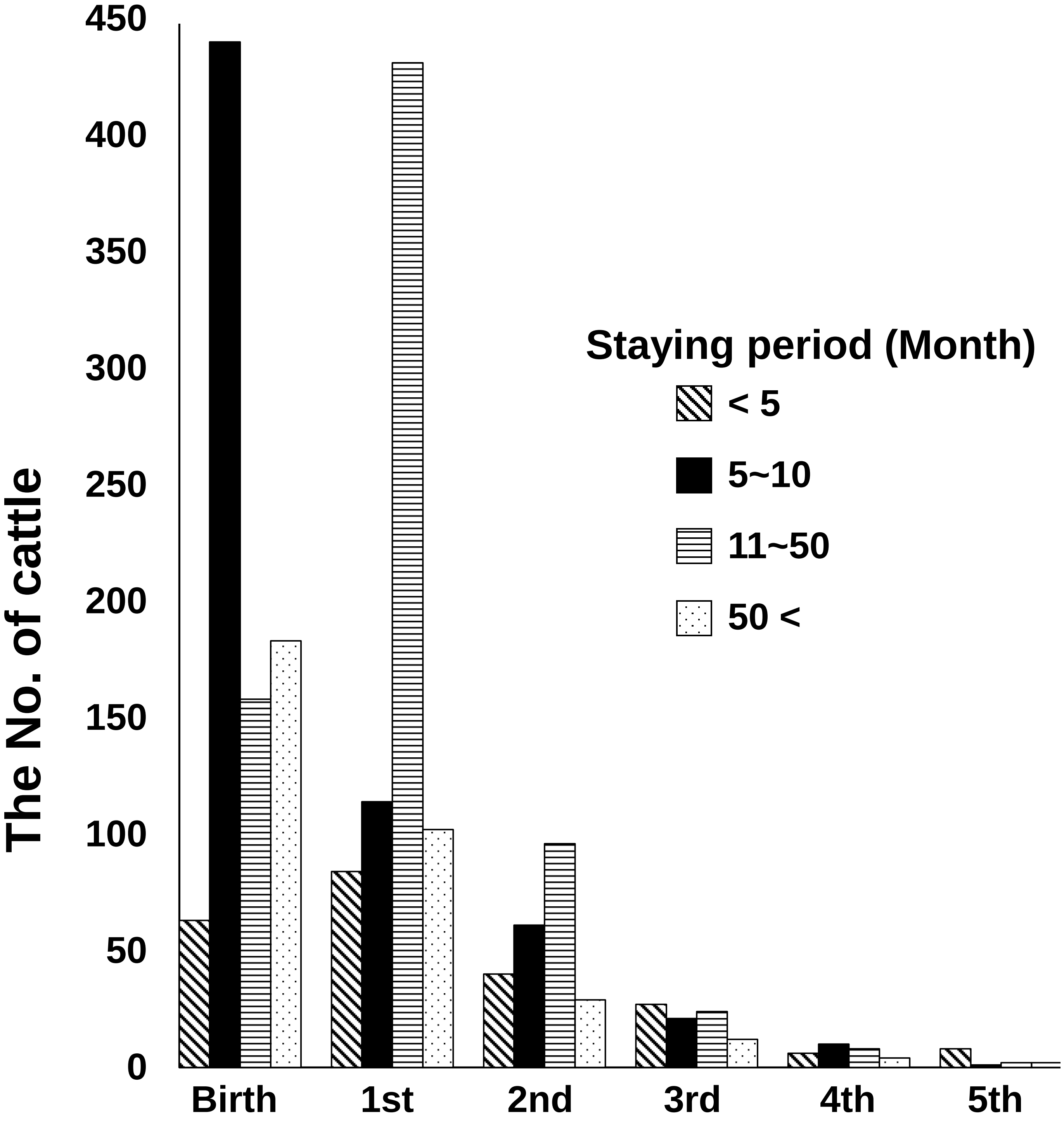

Figure S3. The number of cattle by staying period in each movement. The bar plot indicates the number of cattle by staying period on the farms. The staying period on their birth farms, 1st moved farms, 2nd moved farms, 3rd moved farms, 4th moved farms, and fifth moved farms during movements are shown.
